# Analysis of risk factors for vascular dementia following thrombolytic therapy in patients with acute ischemic stroke

**DOI:** 10.1097/MD.0000000000046905

**Published:** 2025-12-26

**Authors:** Jiejun Wang

**Affiliations:** a Department of Cerebral Vascular Disease I, Neurological Disease Center, Beijing Anzhen Hospital, Capital Medical University, Beijing, China.

**Keywords:** acute ischemic stroke, D-dimer, platelet-to-lymphocyte ratio, recombinant tissue plasminogen activator, risk factors, systemic immune-inflammation index, vascular dementia

## Abstract

This retrospective study investigated the risk factors associated with the development of vascular dementia (VaD) following intravenous thrombolytic therapy in patients with acute ischemic stroke. A total of 219 patients treated between January 2021 and December 2024 were included, comprising 51 individuals who developed VaD during follow-up and 168 without VaD. Diagnosis of VaD was established using the National Institute of Neurological Disorders and Stroke–Association Internationale pour la Recherche et l’Enseignement en Neurosciences criteria. Baseline demographic, clinical, laboratory, and imaging data were systematically collected, including vascular risk factors, metabolic indices, inflammatory and immune markers, and lesion characteristics. Univariate analysis demonstrated that smoking, coronary artery disease, hypertension, and alcohol consumption were significantly more prevalent in the VaD group. Laboratory parameters, including elevated blood glucose, Systemic Immune-Inflammation Index, neutrophil-to-lymphocyte ratio, platelet-to-lymphocyte ratio, lipoprotein, and D-dimer, were significantly higher among patients with VaD. Imaging findings revealed that severe large-vessel stenosis (≥50%) and infarction involving key cognitive-related regions were strongly associated with VaD. Multivariate logistic regression identified smoking history, coronary artery disease, hyperglycemia, elevated Systemic Immune-Inflammation Index, increased platelet-to-lymphocyte ratio, lipoprotein, D-dimer, large-vessel stenosis, and strategic infarct location as independent predictors. These findings underscore the multifactorial etiology of post-thrombolysis cognitive decline and support the need for comprehensive risk stratification and targeted interventions to mitigate VaD risk in acute ischemic stroke patients.

## 1. Introduction

Acute ischemic stroke (AIS) is a leading cause of disability worldwide, and cognitive sequelae are frequent, ranging from mild poststroke cognitive impairment (PSCI) to frank vascular dementia (VaD). Contemporary statements estimate that up to half of stroke survivors manifest measurable cognitive deficits within the first year, underscoring cognition as a critical determinant of long-term outcome and quality of life.^[1]^ While reperfusion with intravenous thrombolysis using recombinant tissue plasminogen activator (rt-PA) improves functional outcomes by salvaging penumbral tissue, its influence on later cognitive trajectories is heterogeneous and likely modified by vascular, inflammatory, thrombotic, and topographic factors.^[2]^

VaD after AIS is classically attributed to cumulative vascular injury and to “strategic” infarcts involving hubs of memory and executive control (e.g., hippocampus, thalamus, basal ganglia, and frontal association cortices). Recent pooled and narrative syntheses reaffirm the prognostic salience of infarct topology for PSCI, while also emphasizing interactions with systemic physiology.^[3]^ Large-artery disease may potentiate cognitive decline through hypoperfusion and microembolization; reviews focused on carotid stenosis describe associations with attention, processing speed, and memory, and suggest that revascularization can improve select domains in carefully characterized cohorts. Beyond macrovascular anatomy, systemic inflammation and hemostasis appear to shape cognitive outcomes. The Systemic Immune-Inflammation Index (SII), neutrophil-to-lymphocyte ratio (NLR), and platelet-to-lymphocyte ratio (PLR) capture leukocyte–platelet activation and have been linked to adverse neurological and cognitive endpoints after stroke. In prospective and cross-sectional studies, higher SII at admission correlates with PSCI, supporting biologic plausibility for immune-vascular coupling in cognitive decline.^[4]^ Parallel lines of evidence implicate infection and inflammation as determinative exposures in poststroke dementia risk trajectories, reinforcing the rationale to integrate inflammatory markers into risk stratification.^[5]^ Pro-thrombotic biomarkers also merit attention: D-dimer levels in AIS vary with onset-to-sampling time and clinical context, and higher values track with worse outcomes, consistent with a role for diffuse fibrin turnover and microthrombosis in ongoing brain injury.^[6]^

Reperfusion timing and completeness may further modify cognitive endpoints. A recent substudy of the AcT randomized trial reported that shorter onset-to-needle times after alteplase or tenecteplase were associated with better 90-day cognitive performance, suggesting that rapid thrombolysis may confer cognitive benefit in addition to functional gains.^[7]^ Endovascular therapy has likewise been associated with favorable performance on multiple cognitive tests in trial populations, highlighting the principle that tissue and network rescue can translate to cognition.^[8]^ At the same time, large cohort analyses indicate that PSCI and poststroke dementia reflect a confluence of traditional vascular risks, stroke-related factors, and systemic biology, arguing for multidomain models rather than single-marker approaches.^[9]^ Machine-learning studies echo this view, prioritizing cortical and strategic infarcts, mesial temporal atrophy, stroke severity, and fasting glucose as high-value predictors.^[10]^

Against this background, the present study investigates determinants of VaD among patients with AIS treated with intravenous thrombolysis. Using standardized diagnostic criteria (NINDS–AIREN) and systematic ascertainment of clinical histories (e.g., smoking and coronary artery disease [CAD]), metabolic parameters (blood glucose and lipoproteins), inflammatory and thrombotic indices (SII, PLR, and D-dimer), and imaging features (large-vessel stenosis severity and strategic region involvement), we sought to identify independent predictors of VaD following reperfusion. Clarifying these associations has practical implications: it may enable early identification of high-risk patients at the time of thrombolysis, inform targeted secondary prevention, and motivate prospective trials of inflammation- and thrombosis-modulating strategies to preserve cognition after stroke.

## 2. Methods

### 2.1. Study design

This study was approved by the Ethics Committee of Beijing Anzhen Hospital (Approval No.: 2020-KY-123, Date: December 15, 2020). This retrospective study included patients with AIS who were treated at our institution between January 2021 and December 2024. Eligible participants were required to be ≥ 18 years of age at the time of admission, have a diagnosis of AIS confirmed by cranial computed tomography or magnetic resonance imaging in accordance with the American Heart Association/American Stroke Association criteria, and have received intravenous thrombolytic therapy with rt-PA within the recommended therapeutic window. Only patients with complete baseline clinical data, including demographic characteristics, vascular risk factors, stroke severity, and laboratory results, as well as a minimum follow-up period sufficient for cognitive assessment and the diagnosis of VaD based on internationally recognized criteria (e.g., NINDS–AIREN), were included.

Patients were excluded if they had a history of preexisting dementia or other neurodegenerative diseases (such as Alzheimer disease, Parkinson disease with dementia, or frontotemporal dementia), severe psychiatric illness, substance abuse, or other conditions precluding reliable cognitive evaluation. Additional exclusion criteria included intracranial hemorrhage, subarachnoid hemorrhage, or primary brain tumors at admission; previous thrombolytic therapy, mechanical thrombectomy, or neurosurgical intervention for the index stroke; and severe comorbidities such as end-stage renal disease, advanced hepatic failure, or uncontrolled malignancy that could compromise survival or follow-up. Based on the occurrence of VaD during follow-up, patients were categorized into the VaD group (n = 51) and the non-VaD group (n = 168). This study was conducted in accordance with the ethical principles of the Declaration of Helsinki and was approved by the institutional ethics committee. Written informed consent was obtained from all participants or their authorized representatives.

### 2.2. Diagnostic criteria for VaD

The diagnosis of VaD was based on the National Institute of Neurological Disorders and Stroke–Association Internationale pour la Recherche et l’ Enseignement en Neurosciences (NINDS–AIREN) criteria.^[11]^ Cognitive function was evaluated by 2 neurologists who had received standardized training and were blinded to the patients’ clinical data and imaging results. Any discrepancies in diagnosis were resolved through discussion with a senior neurologist to reach a consensus.

Patients were classified as having VaD if they met all of the following conditions:

1. Presence of dementia, defined as impairment in memory and at least 2 other cognitive domains (e.g., executive function, attention, language, and visuospatial ability), confirmed by standardized neuropsychological assessment and sufficient to interfere with daily living activities.
2. Cerebrovascular disease evidence, demonstrated by focal neurological signs consistent with stroke and confirmed by neuroimaging (computed tomography or magnetic resonance imaging showing multiple infarctions, strategic single infarcts, or extensive white matter lesions).
3. Temporal relationship between a documented cerebrovascular event and the onset of cognitive decline, or a stepwise and fluctuating course of cognitive deterioration.

Patients were excluded from a diagnosis of VaD if they had clinical or imaging evidence strongly suggestive of primary neurodegenerative disorders, such as Alzheimer disease, Parkinson disease dementia, or frontotemporal dementia.

### 2.3. Data collection

Clinical data potentially associated with the development of VaD following thrombolytic therapy in patients with AIS were systematically collected. Baseline demographic and clinical characteristics included sex, age, body mass index, smoking history, alcohol consumption, history of CAD, history of hypertension, baseline National Institutes of Health Stroke Scale (NIHSS) score prior to thrombolysis, and the therapeutic benefit of thrombolysis. Laboratory parameters obtained at admission comprised blood glucose, hemoglobin, serum creatinine, blood urea nitrogen, total cholesterol, lipoprotein levels, D-dimer, as well as inflammatory and immune indices, including the SII, NLR, and PLR. Imaging data were also recorded, encompassing trial of Org 10172 in acute stroke treatment (TOAST) classification, degree of stenosis and distribution of the responsible large vessels, and whether the infarct lesion involved key cognitive-related regions. Thrombolytic benefit was defined as a reduction of ≥ 4 points in the NIHSS score at 24 hours compared with onset. Infarction involving key regions was defined as lesions affecting at least one cortical area (frontal, temporal, or occipital lobes) or subcortical structures (hippocampus, internal capsule, fornix, caudate nucleus, or thalamus).

### 2.4. Statistical analysis

All statistical analyses were performed using SPSS software, version 28.0 (IBM Corp., Armonk). Categorical variables were expressed as counts and percentages n (%), and comparisons between groups were conducted using the chi-square (χ²) test. Continuous variables were presented as mean ± standard deviation, and group comparisons were assessed using the independent-samples *t* test. Univariate analyses were initially performed to identify potential risk factors associated with the development of VaD following thrombolytic therapy. Variables with statistical significance in univariate analysis were subsequently entered into a multivariate logistic regression model to determine independent predictors. Odds ratios (ORs) with corresponding 95% confidence intervals (CIs) were calculated. Multicollinearity among independent variables was assessed using the variance inflation factor (VIF), with a VIF > 10 indicating severe collinearity. A two-sided *P* value < .05 was considered statistically significant.

## 3. Results

### 3.1. Baseline characteristics

A total of 219 patients with AIS who underwent thrombolytic therapy were included, comprising 51 patients in the VaD group and 168 patients in the non-VaD group. Baseline demographic and clinical characteristics are summarized in Table 1. No significant differences were observed between the 2 groups in terms of sex distribution, age, body mass index, pre-thrombolysis NIHSS score, hemoglobin, serum creatinine, blood urea nitrogen, or total cholesterol levels (all *P* > .05). Similarly, the distribution of TOAST subtypes and responsible vessel locations did not differ significantly between groups (*P* > .05; Table 1).

**Table 1:** Baseline clinical, laboratory, and imaging characteristics of patients with and without vascular dementia following thrombolytic therapy.

Variable	VaD group (n = 51)	Non-VaD group (n = 168)	χ²/t value	*P* value
Demographic and clinical characteristics				
Sex (Male/Female) [n(%)]	29/22	84/84	0.738	.390
Age (yr, ±s)	62.54 ± 8.70	63.59 ± 9.43	−0.740	.461
BMI (kg/m², ±s)	21.19 ± 2.01	21.67 ± 1.93	−1.507	.136
Smoking history, Yes [n(%)]	30 (58.8)	9 (5.4)	76.410	<.001
Drinking history, Yes [n(%)]	20 (39.2)	27 (16.1)	12.433	<.001
CAD history, Yes [n(%)]	26 (51.0)	14 (8.3)	47.664	<.001
Hypertension, Yes [n(%)]	36 (70.6)	43 (25.6)	34.345	<.001
Pre-thrombolysis NIHSS (±s)	18.21 ± 6.38	18.64 ± 5.77	−0.431	.668
Thrombolytic benefit, Yes [n(%)]	17 (33.3)	18 (10.7)	14.907	<.001
Laboratory parameters at admission				
Blood glucose (mmol/L, ±s)	6.75 ± 0.95	5.89 ± 0.98	5.620	<.001
Hemoglobin (g/L, ±s)	141.25 ± 7.51	141.63 ± 7.48	−0.317	.752
Creatinine (μmol/L, ±s)	76.53 ± 7.76	77.42 ± 10.35	−0.660	.511
BUN (mmol/L, ±s)	5.88 ± 0.43	5.81 ± 0.49	0.985	.327
Total cholesterol (mmol/L, ±s)	5.16 ± 0.54	5.26 ± 0.67	−1.092	.278
Lipoprotein (mg/L, ±s)	239.14 ± 82.16	196.72 ± 68.44	3.351	.001
D-dimer (mg/L, ±s)	2.21 ± 0.66	1.71 ± 0.30	5.248	<.001
SII (±s)	585.42 ± 121.87	510.18 ± 123.44	3.850	<.001
NLR (±s)	2.95 ± 0.61	2.70 ± 0.59	2.583	.012
PLR (±s)	68.42 ± 10.62	63.95 ± 9.65	2.688	.009
Imaging data				
TOAST subtype [n(%)]			1.882	.597
Large-artery atherosclerosis	28 (54.9)	105 (62.5)		
Cardioembolism	15 (29.4)	47 (28.0)		
Lacunar/small-vessel occlusion	6 (11.8)	11 (6.5)		
Other	2 (3.9)	5 (3.0)		
Responsible vessel stenosis ≥ 50% [n(%)]	34 (66.7)	28 (16.7)	48.192	<.001
Responsible vessel distribution [n(%)]			4.769	.092
Internal carotid artery	9 (17.6)	37 (22.0)		
Middle cerebral artery	23 (45.1)	94 (56.0)		
Basilar artery	19 (37.3)	37 (22.0)		
Key region infarction, Yes [n(%)]	25 (49.0)	14 (8.3)	44.247	<.001

BMI = body mass index, BUN = blood urea nitrogen, CAD = coronary artery disease, NIHSS = National Institutes of Health Stroke Scale, NLR = neutrophil-to-lymphocyte ratio, PLR = platelet-to-lymphocyte ratio, SII = systemic immune-inflammation index, TOAST = trial of Org 10172 in acute stroke treatment, VaD = vascular dementia.

### 3.2. Univariate analysis of potential risk factors for VaD after thrombolytic therapy

Univariate analysis revealed that patients in the VaD group had a significantly higher prevalence of smoking history (58.8% vs 5.4%, *P* < .001), CAD (51.0% vs 8.3%, *P* < .001), hypertension (70.6% vs 25.6%, *P* < .001), and drinking history (39.2% vs 16.1%, *P* < .001) compared with the non-VaD group. Laboratory parameters showed that blood glucose (6.75 ± 0.95 vs 5.89 ± 0.98 mmol/L, *P* < .001), SII (585.42 ± 121.87 vs 510.18 ± 123.44, *P* < .001), NLR (2.95 ± 0.61 vs 2.70 ± 0.59, *P* = .012), PLR (68.42 ± 10.62 vs 63.95 ± 9.65, *P* = .009), lipoprotein levels (239.14 ± 82.16 vs 196.72 ± 68.44 mg/L, *P* = .001), and D-dimer (2.21 ± 0.66 vs 1.71 ± 0.30 mg/L, *P* < .001) were all significantly higher in the VaD group. Imaging findings demonstrated that patients with VaD were more likely to have responsible large-vessel stenosis ≥ 50% (66.7% vs 16.7%, *P* < .001) and infarction involving key cognitive-related regions (49.0% vs 8.3%, *P* < .001; Table 1).

### 3.3. Multivariate logistic regression analysis identifying independent risk factors for VaD

Variables that were statistically significant in univariate analysis were entered into a multivariate logistic regression model. As shown in Table 2, independent risk factors for the development of VaD following thrombolytic therapy included smoking history (OR = 8.25, 95% CI: 2.95–23.06, *P* < .001), CAD (OR = 5.93, 95% CI: 2.29–15.37, *P* < .001), elevated blood glucose (OR = 1.95, 95% CI: 1.37–2.78, *P* < .001), higher SII (OR = 1.36 per 100 units, 95% CI: 1.13–1.64, *P* = .001), increased PLR (OR = 1.04, 95% CI: 1.01–1.08, *P* = .012), elevated lipoprotein levels (OR = 1.01, 95% CI: 1.00–1.02, *P* = .028), elevated D-dimer levels (OR = 3.07, 95% CI: 1.55–6.09, *P* = .001), responsible large-vessel stenosis ≥ 50% (OR = 7.11, 95% CI: 2.47–20.44, *P* < .001), and infarction involving key regions (OR = 10.41, 95% CI: 3.48–31.12, *P* < .001; Table 2).

**Table 2:** Multivariate logistic regression analysis of risk factors for vascular dementia after thrombolytic therapy in acute ischemic stroke patients.

Variable	β	SE	Wald	OR	95% CI for OR	*P* value
Smoking history (Yes vs No)	2.11	0.52	16.46	8.25	2.95–23.06	<.001
CAD history (Yes vs No)	1.78	0.49	13.22	5.93	2.29–15.37	<.001
Blood glucose (mmol/L)	0.67	0.18	13.86	1.95	1.37–2.78	<.001
SII (per 100 units)	0.31	0.10	9.61	1.36	1.13–1.64	.001
PLR	0.04	0.02	6.28	1.04	1.01–1.08	.012
Lipoprotein (mg/L)	0.01	0.004	4.85	1.01	1.00–1.02	.028
D-dimer (mg/L)	1.12	0.35	10.24	3.07	1.55–6.09	.001
Responsible vessel stenosis ≥ 50%	1.96	0.54	13.16	7.11	2.47–20.44	<.001
Key region infarction (Yes vs No)	2.34	0.56	17.48	10.41	3.48–31.12	<.001

CAD = coronary artery disease, CI = confidence interval, OR = odds ratio, PLR = platelet-to-lymphocyte ratio, SE = standard error, SII = systemic immune-inflammation index.

### 3.4. Multicollinearity diagnostics of independent variables using VIF

Multicollinearity among the independent variables included in the multivariate logistic regression model was assessed using the VIF. The analysis demonstrated that all variables exhibited VIF values ranging from 1.3 to 1.7, with corresponding tolerance values above 0.5. These results indicated that none of the predictors exceeded the commonly accepted threshold for multicollinearity (VIF > 5 for moderate or VIF > 10 for severe collinearity). Therefore, no significant collinearity was present, and the stability and reliability of the logistic regression model were not adversely affected.

## 4. Discussion

This study identified a profile of clinical, laboratory, and imaging factors independently associated with the development of VaD after intravenous thrombolysis for AIS. Smoking history, CAD, higher admission glucose, elevated SII, increased PLR, higher lipoprotein levels, elevated D-dimer, responsible large-vessel stenosis ≥ 50%, and infarction involving key cognitive-related regions were independently associated with poststroke VaD. These findings suggest that both preexisting macrovascular burden and the acute inflammatory-thrombotic response to cerebral ischemia contribute to subsequent vascular cognitive impairment.

Smoking and CAD reflect cumulative atherosclerotic exposure and endothelial dysfunction. Their independent associations with VaD in our cohort are biologically plausible given established links between macrovascular disease, cerebral hypoperfusion, embolic load, and small-vessel injury that collectively degrade network efficiency required for memory and executive function. Elevated admission glucose likely indexes stress hyperglycemia and chronic dysglycemia; both amplify ischemia–reperfusion injury through oxidative stress and microvascular dysfunction, which are implicated in poststroke cognitive decline. The independent signals carried by SII and PLR indicate that leukocyte–platelet activation at presentation is not an epiphenomenon but an active component of the cascade linking acute vascular injury to longer-term cognitive outcomes. Higher lipoprotein concentrations may act through atherogenic and pro-inflammatory pathways that potentiate white-matter damage and lacunar disease. Elevated D-dimer supports a role for ongoing fibrin turnover and a pro-thrombotic milieu that may sustain microembolization or impair microcirculatory flow, thereby enlarging strategic or diffuse ischemic injury. The strong associations for ≥ 50% stenosis in the responsible artery and for infarction in key cognitive regions emphasize the joint importance of upstream hemodynamic constraints and lesion topology in determining cognitive prognosis.

The absence of group differences in age, baseline NIHSS, creatinine, hemoglobin, blood urea nitrogen, total cholesterol, TOAST subtype distribution, and arterial territory location suggests that, within a thrombolysed AIS population meeting standard eligibility, vascular and inflammatory signatures, rather than global demographics or gross stroke classifications, may more precisely stratify VaD risk. Together, our data support a multidomain risk architecture in which modifiable macrovascular factors (smoking, CAD, and stenosis), acute immune-thrombotic markers (SII, PLR, and D-dimer), metabolic state (glucose), and lesion topology (strategic regions) jointly determine post-thrombolysis cognitive trajectories.

Recent work has refined understanding of cognitive outcomes after reperfusion therapy. A 2024 systematic review and meta-analysis of reperfusion in patients with dementia or cognitive impairment reported that intravenous thrombolysis and endovascular therapy remain effective and safe in this population, arguing against excluding cognitively vulnerable patients from acute treatment pathways; however, the review also underscored the need to characterize cognitive outcomes longitudinally after reperfusion.^[12]^ Complementing this, a 2024 registry analysis found that preexisting dementia did not diminish the efficacy or safety of thrombolysis or thrombectomy, reinforcing guideline-concordant use of rt-PA while motivating research into posttreatment cognition.^[13]^ Our data extend these observations by focusing on determinants of VaD after thrombolysis and by quantifying the contributions of inflammatory and thrombotic indices (SII, PLR, and D-dimer) alongside vascular anatomy. Inflammation-based hematologic markers have gained attention as correlates of PSCI. A 2025 meta-analysis reported higher NLR and PLR among patients with PSCI than in those without, supporting an association between peripheral inflammatory tone and cognitive sequelae.^[14]^ Beyond PSCI, 2024 work linked SII to dementia severity in Alzheimer disease and to vascular disease risk, suggesting that SII captures a generalizable immune-vascular signal relevant to cognitive decline.^[15]^ Our finding that SII and PLR independently predict VaD aligns with these reports and indicates applicability in a thrombolysed AIS cohort.

Cerebrovascular stenosis and lesion topology are also central. Severe carotid stenosis has been associated with cognitive deficits and, in interventional series, cognitive improvement after revascularization, consistent with hypoperfusion mechanisms.^[16]^ With respect to lesion location, recent reviews emphasize “strategic” territories – thalamus, hippocampus/medial temporal lobe, caudate/basal ganglia, and frontal association cortex – as high-yield predictors of PSCI.^[17]^ Our operational definition of key cognitive-related regions is concordant with these summaries and our results demonstrate a strong, independent effect of such involvement on VaD risk. Finally, accumulating evidence suggests that thrombolysis timing influences cognitive endpoints. A 2025 analysis reported that shorter onset-to-needle times were associated with higher Montreal Cognitive Assessment scores and lower cognitive impairment at 90 days, proposing cognitive benefit from faster reperfusion.^[7]^ While our study did not capture treatment timing granularity, the observed protective association of “thrombolytic benefit” (≥4-point NIHSS reduction) with lower VaD risk is directionally consistent with this literature.

The observed pattern of risk factors supports a mechanistic framework in which high atherothrombotic load (smoking, CAD, stenosis, and lipoprotein levels) interacts with acute systemic inflammation and platelet activation (SII and PLR) to amplify microvascular obstruction and endothelial injury during and after ischemia. Elevated SII and PLR reflect increased neutrophil- and platelet-mediated immune activation, which can trigger release of cytokines, reactive oxygen species, and matrix metalloproteinases, leading to blood–brain barrier disruption, endothelial dysfunction, and impaired microcirculatory perfusion. Activated platelets also promote leukocyte adhesion and microthrombus formation within small vessels, further aggravating hypoperfusion in cognitively relevant regions.

Elevated D-dimer suggests persistent fibrin turnover and may indicate ongoing microthrombi formation or breakdown, potentially sustaining diffuse ischemic stress in watershed and periventricular white matter even after large-vessel reperfusion. This pro-thrombotic and pro-inflammatory milieu contributes to secondary neuroinflammation and chronic endothelial damage. Hyperglycemia can potentiate leukocyte adhesion, blood–brain barrier dysfunction, and oxidative stress, further limiting neuronal recovery.

When infarction extends into strategic hubs involved in memory encoding (hippocampus), thalamo-cortical relay, or frontostriatal executive circuits, the threshold for clinical VaD is more readily crossed, even if overall infarct volume is modest. Therefore, the convergence of endothelial injury, neuroinflammation, microvascular thrombosis, and strategic infarction after thrombolysis may identify a subgroup at high risk for vascular cognitive impairment despite initial reperfusion.

Limitations warrant consideration. First, the retrospective single-center design may introduce selection bias and restrict causal inference. Detailed pre-stroke cognitive status or cognitive reserve could not be systematically evaluated, and although patients with known dementia or neurodegenerative diseases were excluded, the presence of subclinical cognitive impairment cannot be completely ruled out. Second, despite adjusting for major vascular, metabolic, inflammatory, and imaging-related variables, residual confounding from unmeasured factors (such as small vessel disease burden, socioeconomic status, education level, or mood disorders) remains possible. Some of these variables were not consistently available and thus were not included in the multivariable model. Third, onset-to-needle time and reperfusion grade were not recorded in detail, preventing evaluation of time-dependent cognitive benefits of thrombolysis reported in previous studies. Biomarkers were measured only at admission; dynamic changes in SII, PLR, and D-dimer over time might provide better prognostic value. Neuroimaging markers of cerebral small vessel disease (e.g., white matter hyperintensities, lacunes, and microbleeds) were not quantitatively assessed, which may limit mechanistic insights. Furthermore, cognitive outcomes were classified dichotomously as VaD rather than assessed across cognitive domains, which may overlook milder forms of impairment. Finally, external validation in larger, multicenter cohorts and under different thrombolytic protocols (including tenecteplase) is necessary to confirm generalizability.

## 5. Conclusions

In this retrospective analysis of patients with AIS undergoing thrombolytic therapy, smoking history, CAD, elevated blood glucose, SII, PLR, lipoprotein, D-dimer, responsible large-vessel stenosis, and infarction involving cognitive-related regions were identified as independent predictors of VaD. These findings highlight the multifactorial nature of post-thrombolysis cognitive decline and provide evidence for risk stratification and early targeted intervention to preserve cognitive outcomes.

## Author contributions

**Conceptualization:** Jiejun Wang.

**Data curation:** Jiejun Wang.

**Formal analysis:** Jiejun Wang.

**Funding acquisition:** Jiejun Wang.

**Investigation:** Jiejun Wang.

**Writing – original draft:** Jiejun Wang.

**Writing – review & editing:** Jiejun Wang.
